# Advances in autogenous dentin matrix graft as a promising biomaterial for guided bone regeneration in maxillofacial region: A review

**DOI:** 10.1097/MD.0000000000039422

**Published:** 2024-08-23

**Authors:** Honglan Sun, Xiaoyunqing Yin, Chao Yang, Huifang Kuang, Wen Luo

**Affiliations:** a Key Laboratory of Emergency and Trauma of Ministry of Education, Department of Stomatology, Key Laboratory of Hainan Trauma and Disaster Rescue, The First Affiliated Hospital of Hainan Medical University, Haikou, Hainan Province, China; b School of Stomatology, Hainan Medical University, Haikou, Hainan Province, China; c Department of Stomatology, The People’s Hospital of Longhua, Shenzhen, Guangdong Province, China; d Research and Development Department, Shenzhen Uni-medica Technology Co., Ltd, Shenzhen, Guangdong Province, China.

**Keywords:** autogenous dentin matrix, bone substitutes, clinical applications, dentin matrix graft, guided bone regeneration

## Abstract

Autogenous dentin matrix (ADM), derived from a patient’s extracted tooth, can be repurposed as an autologous grafting material in reconstructive dentistry. Extracted teeth provide a source for ADM, which distinguishes itself with its low rejection rate, osteoinductive capabilities and ease of preparation. Consequently, it presents a viable alternative to autogenous bone. Animal studies have substantiated its effective osteoinductive properties, while its clinical applications encompass post-extraction site preservation, maxillary sinus floor augmentation, and guided bone tissue regeneration. Nevertheless, the long-term efficacy of ADM applied in bone regeneration remains underexplored and there is a lack of standardization in the preparation processes. This paper comprehensively explores the composition, mechanisms underlying osteoinductivity, preparation methods, and clinical applications of ADM with the aim of establishing a fundamental reference for future studies on this subject.

## 1. Introduction

The field of oral implantology has emerged as a reliable and well-documented technique for effectively treating edentulism, offering long-term functional and esthetic benefits.^[1]^ However, edentulism frequently leads to the loss of alveolar ridge, thereby complicating periodontal and implant surgeries, restorative treatments, as well as orthodontic procedures.^[2]^ To address this issue, various bone grafting materials are employed for the restoration or enhancement of the alveolar ridge bone. Autogenous bone grafts, considered as the gold standard, provide osteogenesis, osteoinduction, and osteoconduction capabilities. However, their utilization is constrained by complications associated with donor sites, potential additional trauma risks, and limited availability of harvested bone quantities.^[3,4]^

Alternative grafting materials, such as allogeneic, xenogeneic, and biological substitutes, have emerged as potential alternatives to autologous bone grafts; however, they present challenges including increased infection risks, unpredictable bone resorption rates, prolonged healing durations, and higher associated costs.^[5]^ Bone graft and substitute materials currently used in the dental field have been classified and expounded (as shown in Table 1). Based on the unique characteristics of various bone graft materials, they are utilized to treat specific types of bone defects. Ideally, bone graft materials should stabilize alveolar socket blood clots, provide a robust biomechanical scaffold for osteogenic cells, encompass osteogenic growth factors, and exhibit a balanced resorption rate and bone formation remodeling.^[21–23]^

**Table 1:** The main characteristics of various bone grafts.

Graft	Definition	Advantages	Disadvantages	Examples	References
Autogenous bone graft	Bone from the patient’s own body	• High osteogenic potential • Excellent biocompatibility • No risk of transmission	• Need for second surgical site • Limited amount of graft material	Mandibular, cortical bone, chin, iliac crest	[6–8]
Allograft	Bone from different individual	• Wide availability • Avoidance of donor site morbidity • Preservation of biologic properties	• Potential for disease transmission • Lower osteogenic potential than autograft	FDBA, DBA	[9–11]
Xenograft	Grafts derived from a genetically different species than the recipient	• Architecture and geometric structure resemble bone • No need of additional surgery • Some grafts have excellent bone conductivity • Well-documented	• Processed extensively to remove viable cells and biological components • Resorption rate is highly variable • Possible disease transmission and potential unwanted immune reactions	Bovine bone graft, porcine bone graft, red algae	[12,13]
Alloplastic bone graft	Fabricated bone substitutes	• Readily available • Controlled properties • No disease transmission	• Not predictable absorption • Lack osteogenic potential	HA, TCP, calcium sulfate, bioactive glasses, NiTi, PMMA	[14]
Synthetic bioceramics	Bone substitutes with infused growth factor or living osteogenic cells	• High bioactivity • Osteoinductive properties • Chemical similarity with bone • Stimulation of osteoblast growth	• High cost • Not predictable absorption	Sticky bone (PRF added), Osigraft (BMP-7 added), infuse bone graft (rhBMP-2 added)	[15–17]
Autogenous dentin graft	Processed dentin from the patient’s own extracted teeth	• Biocompatible • Osteoconductive and osteoinductive • No disease transmission • No rejection risk • High similarity with human cortical bone	• Not suitable for large defects • Requires additional processing time • Cleaning and sterilization process partially alters biological performances • Limited long-term clinical data	DDM, MDM, dental particles or granules dental powder	[18–20]

DBA = demineralized bone allograft, DDM = demineralized dentin matrix, FDBA = freezed-dried bone allograft, HA = hydroxyapatite, MDM = mineralized dentin matrix, PMMA = polymethyl methacrylate, PRF = platelet-rich fibrin, rhBMP = recombinant human bone morphogenetic protein, TCP = tricalcium phosphate.

The autogenous dentin matrix (ADM), derived from discarded teeth, exhibits compositional similarities with alveolar bones and effectively addresses various limitations associated with other grafting materials.^[24]^ The use of ADM grafts eliminates the potential risks associated with allografts and xenografts, such as cross-contamination, immunogenicity, and donor variability. Additionally, ADM obviates the need for a secondary harvesting site, resulting in decreased morbidity and lower rates of graft resorption compared to bone autografts.^[25–28]^

The ADM encompasses a multitude of growth factors that are indispensable for osteogenesis, demonstrating both osteoconductive and osteoinductive capabilities.^[29,30]^ Following a series of procedures including thorough cleaning, dehydration, demineralization, and sterilization, autologous dentin can be finely ground to the desired dimensions and effectively utilized for clinical bone augmentation.^[31]^ Currently, its application in bone augmentation techniques is both successful and advancing, positioning it as a commendable alternative to autogenous bone grafts.^[32,33]^ While the suitability of a graft material often hinges on its intended application, this review delves into the histological composition, mechanisms underlying osteoinductivity, preparation process, and clinical applications of ADM (as shown in Table 2), aiming to lay a foundational reference for future ADM studies.

**Table 2:** Clinical research reports of ADM.

Clinical application	Author and year	Included number of people	Research groups	Follow-up	Healing evaluation method	Result
Alveolar ridge preservation	Elfana et al^[34]^ 2021	Experimental group: 10 cases Control group: 10 cases	Experimental group: AWTG Control group: ADDG	6 mo	CBCT	AWTG and ADDG are similarly effective in alveolar ridge preservation. Histologically ADDG seems to demonstrate better graft remodeling, integration and osteoinductive properties.
Maxillary sinus floor augmentation	Jun et al^[35]^ 2014	Experimental group: 22 cases Control group: 21 cases	Experimental group: AutoBT Control group: Bio-Oss	4 mo	MicroCT	AutoBT could be considered a viable alternative to the autogenous bone or other bone graft materials in sinus bone graft procedure.
Immediate implantation	Issa et al^[36]^ 2024	Group I: 13 cases Group II: 13 cases Group III: 13 cases	Group I: without grafting. Group II: ATBG. Group III: Simvastatin gel mixed with ATBG.	6 mo, 12 mo	CBCT	ATBG has been successfully utilized for immediate implant placement in fresh sockets with labial bone defects. ATBG with simvastatin in periodontally compromised sites could improve implant osseointegration and promote favorable changes in peri-implant tissues.
Guided bone regeneration	Li et al^[37]^ 2018	Experimental group: 22 cases Control group: 21 cases	Experimental group: DDM Control group: Bio-Oss	6 mo, 18 mo		The autogenous DDM granules prepared at the chairside after extractions could act as an excellent readily available alternative to bone graft material in GBR, even for implantation of severe periodontitis cases.
Combination with other materials	Yüceer-Çetiner et al^[38]^ 2021	Group D: 20 samples Group DP: 21 samples Group C: 16 samples	Group D: autogenous dentin graft Group DP: autogenous dentin graft and PRF Group C: empty	3 mo	Histological and immunohistochemical evaluations, scanning electron microscopy	Undemineralized autogenous dentin graft has bone formation capacity on early period of bone healing. It can be used as bone graft material in augmentation procedures and its combined use with PRF accelerates new bone formation.

ADDG = versus autogenous demineralized dentin graft, ADM = autogenous dentin matrix, ATBG = autogenous tooth bone graft, AutoBT = autogenous tooth bone graft material, AWTG = autogenous whole tooth, CBCT = cone-beam computed tomography, DDM = demineralized dentin matrix, IIP = immediate implant placement, PRF = platelet-rich fibrin.

## 2. Histologic composition

The composition of autogenous dentin closely resembles that of autogenous bone, primarily comprising organic components (20%), nonorganic components (70–75%), and water (10%).^[39]^ Its significantly reduced fat content and exclusion of bone marrow constituents facilitate its preparation for bone grafting.^[40]^

### 2.1. Organic components

The organic component of dentin is predominantly composed of type I collagen fibers, accounting for approximately 90%.^[41]^ These fibers play an important role in calcification. The trimeric superhelical collagen structure facilitates the deposition of mineralized crystals, attachment of biological factors, and effectively supports bone regeneration by positively influencing osteoblastic cell responses.^[18]^ In addition to collagen, dentin comprises non-collagenous proteins (NCPs), proteoglycans, carbohydrate, lipids, etc.^[42]^ NCPs include phosphoproteins and Non-phosphoproteins.

#### 2.1.1. Phosphoproteins

The proteins in this category include sibling proteins, namely dentin phosphoprotein (DPP), dentin sialoprotein (DSP), osteobontin, and osteonectin. Notably, DPP and DSP play a crucial role in the mineralization and crystallization of collagen fibers, initiating the process of osteogenesis that stimulates bone resorption.^[43–45]^

#### 2.1.2. Non-phosphoproteins

This group comprises osteocalcin, a calcium-binding protein, and crucial growth factors such as transforming growth factor (TGF), bone morphogenetic proteins (BMPs), vascular endothelial growth factor (VEGF), insulin-like growth factor (IGF), fibroblast growth factor (FGF), platelet-derived growth factor (PDGF) and other significant growth factors. Among these, TGF-β1 has been identified as a pivotal growth factor that synergistically promotes osteoblast differentiation with BMPs.^[46–48]^ Both TGF-β1 and BMPs are capable of independently activating the RunX2 pathway to induce osteogenesis.^[49,50]^ TGF-β1 also functions as a synergistic signaling molecule in conjunction with other growth factors, such as FGF and IGF.^[51,52]^ The signaling networks, which play a crucial role in the migration of mesenchymal stem cells (MSCs) and macrophages to the wound site, also induce significant MSCs and macrophage migration to promote wound healing. Additionally, these networks stimulate MSC proliferation and enhance collagen-like bone matrix production. Other synergistic signaling pathways were observed in ADM. Moreover, TGF-1 was identified as the most abundant growth factor, surpassing BMP-2, FGF, PDGF, and VEGF by more than twofold. Notably, VEGF is recognized as a pivotal mitogen that regulates neo endothelial cell outgrowth for hemotransfusion, and the pro-angiogenic effects of VEGF were potentiated by the synergistic actions of PDGF and FGF.^[53,54]^ Avery et al also highlighted the significance of considering the synergistic impact of growth factors on bone formation in ADM.^[54]^ It is crucial to acknowledge that these growth factors not only effectively promote the proliferation of new endothelial cells but also stimulate their osteogenic effects in a synergistic manner. Furthermore, it is essential to emphasize that other matrix proteins, such as disaccharide chain proteoglycans, may act as supplementary adjuvants to enhance bioactivity.^[55]^ Moreover, it has been demonstrated that disaccharide chain proteoglycans possess the ability to directly stimulate bone formation by activating both the BMP/TGF-β and classical Wnt/β-catenin signaling pathways, rendering them a potential therapeutic approach for addressing bone-related disorders.^[56]^

### 2.2. Inorganic mineralized fractions

The inorganic components of dentin primarily consist of apatite crystals, accounting for 70–75%.^[57]^ The crystal structure of tooth roots exhibits a low-crystallinity phosphate structure, which is characteristic of autogenous bone. It has been suggested that the smaller-sized hydroxyapatite (HA) may enhance biodegradation due to its increased solubility.^[58]^ The X-ray diffraction (XRD) analysis conducted by Kim et al^[39,59]^ demonstrated that the crystalline structure, domain size, and Ca/P ion solubility of ADM were comparable to those of autogenous bone calcium phosphate. Various compositions were identified, including HA (Ca/*P* = 1.75), tricalcium phosphate (TCP) (Ca/*P* = 1.46), amorphous calcium phosphate (ACP, Ca/*P* = 1.32), and octacalcium phosphate (OCP, Ca/*P* = 1.24). Priya et al^[60]^ emphasized that acid-etching dissolution of calcium phosphate complexes leads to the liberation of calcium and phosphorus ions, thereby catalyzing the reprecipitation of apatite on the surface. The proposed dissolution-reprecipitation sequence is suggested to underlie the formation of apatite, thereby potentially enhancing osseointegration in bioceramic composites. Furthermore, the authors observed the emergence of macroporous regions in calcium phosphate composites due to expansive dissolution triggered by the a-TCP and CaO phases. Such macroporosity and surface roughness are believed to promote biological cell proliferation and bone growth.^[61]^

Electron microscopic observations reveal that autogenous dental bone graft material exhibits a densely packed microporous structure with low crystallinity. As a result, the density, roughness, and uniformity of autogenous dentin closely resemble those of autogenous cortical bone. Moreover, the collagen network reinforces the blood clot within the alveolar socket, serving as a robust biomechanical scaffold facilitating the migration of osteoblastic cells, it also serves as a reservoir for bone-enhancement proteins such as growth factors, thereby ensuring optimal rates of resorption and remodeling in the bone formation process.^[62,63]^

## 3. Osteogenic mechanism

Upon preopening the bone marrow cavity, stem cells and osteoblasts originating from the bone endosteum become exposed.^[64]^ The newly formed socket is filled with platelet-rich blood, leading to the subsequent encapsulation of the biomaterial’s surface and gaps by blood clots. During the initial phase, platelet degranulation releases various growth factors such as PDGF-AA/AB/BB, TGF-β1, TGF-β2, VEGF, and epidermal growth factor (EGF). Additionally, hyaluronan is released while fibronectin from coagulating plasma in the blood deposits on the surface of the biomaterial.^[65]^ The deposition establishes a connection between the biomaterial and the surface of the bone wall, facilitating interaction. The growth factors released through platelet degranulation stimulate various cells including bone marrow cells, endothelial cells, and osteoblasts present in the endosteum of the bone. These factors promote cell migration, differentiation, angiogenesis, and mitosis.^[66]^

During cell division, a process of “creeping substitution” occurs, wherein daughter cells propel forward while the parent osteoblasts undergo maturation. These mature osteoblasts secrete osteoid and gradually differentiate into bone cells.^[67]^ The continuous occurrence of cell division and creeping substitution ultimately leads to the closure of the gap between the bone wall and the biomaterial.^[68]^

The crux of bone tissue generation lies in the intricate interplay between cells, matrix, and environmental factors, leading to cellular expansion and secretion of matrix molecules.^[65,66]^ An ideal biomaterial for this purpose should exhibit progressive resorption and remodeling capabilities.^[69]^ When considering autogenous dentin as a potential biomaterial for tissue engineering and regenerative medicine, several crucial factors come to the forefront:

### 3.1. Osteoconductivity

The demineralization process of body dentin reveals a cross-linked collagen fiber network, leading to an expansion in the diameter of dentin tubules and the acquisition of appropriate porosity necessary for their function as scaffolds.^[70]^ These optimized scaffolds possess the ability to accommodate cells, coordinate their activities, and present microstructures that facilitate the attachment of cell adhesion molecules derived from blood and platelets.^[71]^ Furthermore, they provide a matrix conducive to recruiting, proliferating, and differentiating bone progenitor cells even prior to resorption.^[72]^ Moreover, autologous dentin matrix can serve not only as a scaffold but also as a carrier for external cells, growth factors, and genes; thus enhancing its versatility and significance in regenerative medicine.^[73,74]^ In a study conducted by Lee et al,^[75]^ the osteogenic potential of demineralized human dentin matrix was evaluated through quantification of MG-63 cell line proliferation and differentiation, in comparison to a composite material consisting of inorganic bovine bone and collagen. Throughout all observed time intervals, cell adhesion and proliferation on the ADM surface were significantly more abundant than those on Bio-Oss collagen (Geistlich, Wolhusen, Switzerland). ADM consistently outperformed Bio-Oss collagen in terms of both cell adhesion and growth at each examined interval. Cells cultured on ADM exhibited not only a more flattened morphology but also displayed a uniform distribution pattern. Insights obtained from confocal laser scanning microscopy emphasized ADM’s superior capability for cytoplasmic spreading attachment. Furthermore, immunofluorescence assays revealed notably higher fluorescence intensity of osteocalcin, an essential biomarker for cell differentiation, on ADM, highlighting its exceptional osteoconductivity.^[76]^

### 3.2. Osteoinductivity

Osteoinductivity is the inherent capacity of a substance to stimulate progenitor cells to differentiate into osteoblasts, which are responsible for bone formation. ADM exemplifies this potential, primarily due to its inclusion of osteogenesis-related growth factors.^[77]^ These factors stimulate osteogenic precursor cells in the host’s connective tissues, directing them towards differentiation and subsequent bone generation.^[78]^ Historical research highlights the osteoinductive ability of ADM. Bessho et al^[79]^ successfully isolated bone morphogenetic proteins (BMPs) from human dentin matrix. Although there are differences between BMPs derived from human dentin and human bone, their in vivo functionality remains comparable as both promote similar outcomes in terms of bone formation.^[79]^ Wang and Ike’s work further emphasized the osteoinductive efficacy of materials derived from human dentin.^[80,81]^ The study suggests that even small fragments of wisdom teeth, typically considered as surgical waste, may be repurposed as bone graft materials due to the inherent osteoinductive properties of dentin.^[82]^ Additionally, noteworthy observations were made by Kim et al^[83–85]^ when transplanting human ADM into the muscular tissue of nude mice. The subsequent emergence of cartilage and bone, along with the observation of newly formed osteoid on ADM granules, attested to its potential in osteoinduction. Nevertheless, Rijal et al^[86]^ expressed a contrasting viewpoint by arguing that the osteoinductive potential of human dentin might not be as evident as suggested by others. However, they acknowledged the non-antigenic properties of ADM which implies that even if its osteoinductive properties are contested, its biocompatibility remains an advantageous feature.

In conclusion, although there exists substantial evidence supporting the osteoinductive capability of ADM, further comprehensive research and controlled studies are imperative to establish a consensus within the scientific community. This debate also underscores the intricate and multifaceted nature of biomaterial research in the field of regenerative medicine.

### 3.3. Biocompatibility

The dentin and bones of the maxilla and mandible both originate from neural crest cells, indicating a shared embryological heritage that results in notably similar compositions. Autologous dentin, when subjected to dehydration, degreasing, and demineralization, exhibits minimal immunogenicity and antigenicity.^[79]^ Moreover, it demonstrates excellent biodegradability and degrades synchronously with new bone formation.^[87]^ Notably, it plays a pivotal role in expediting BMPs release which transforms undifferentiated stromal cells into osteoblasts and odontoblasts while championing bone tissue regeneration – encapsulating the 3 core principles of osteogenesis.^[88]^

The biocompatibility of ADM, combined with its osteoconductive, osteoinductive, and osteogenic properties, renders it a promising alternative to conventional bone graft materials. With further research and elucidation of its benefits, this material holds the potential to supplant bone grafting practices in the dental field.

## 4. Process of preparation of ADM grafts

**Figure 1.**
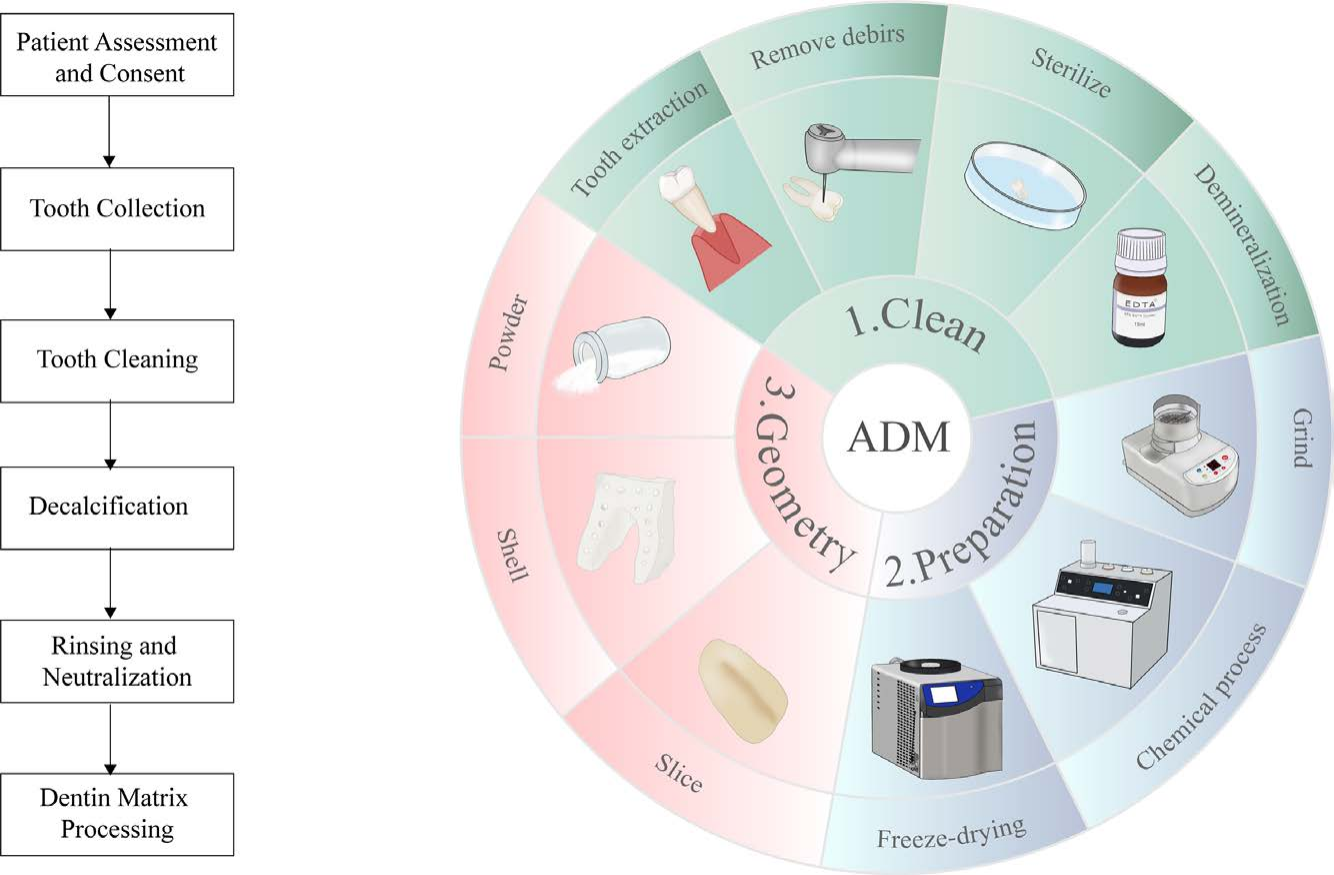
Overview of preparation of ADM grafts. ADM = autogenous dentin matrix.

The transformation of discarded teeth into a biocompatible graft material necessitates a multi-step, meticulous process to ensure both safety and efficacy (as shown in Fig. 1).

### 4.1. Cleaning and pretreatment

Discarded teeth undergo an initial cleansing phase, during which all contaminants and extraneous elements are eliminated. This includes:

- Extraction of the pulp.
- Removal of calculus, surface granulation tissue, and any prior dental restorations or root canal filling materials.
- Thorough rinsing with distilled water to clean off impurities.

### 4.2. Chemical and physical processing

Following the cleaning process, various procedures are conducted on the teeth to optimize their compatibility and prolong their shelf-life:^[89]^

- Dehydration: removes water content to stabilize the dentin.
- Degreasing: rids the dentin of any residual lipids or fats.
- Demineralization: by reducing the mineral content of the tooth, dentin becomes more malleable and biologically active, thereby enhancing its malleability and biological activity. This process leads to the enlargement of dentin tubules, which in turn exposes the underlying collagen network. The augmented exposure ensures prompt release of osteoinductive growth factors, thereby amplifying the graft’s potential for osteogenesis.^[90]^ The osteogenic profile of demineralized dentin is notably superior to that of non-mineralized dentin.
- Calcination: this step entails subjecting the material to controlled thermal conditions, potentially leading to sterilization and modification of its physical properties.
- Lyophilization (freeze drying): the moisture is effectively removed from the dentin, ensuring its long-term preservation without any degradation.
- Ethylene oxide sterilization: a method to ensure the complete eradication of all potential pathogens, thereby ensuring the safety of the graft for implantation.

### 4.3. Storage

The processed and ground graft can be readily utilized in a clinical setting. Alternatively, it can be stored in a specialized solution. Under appropriate storage conditions, the shelf-life of ADM products can exceed 5 years at room temperature.^[91]^

Considering their crucial involvement in cellular processes such as migration, adhesion, and differentiation, the majority of preparation procedures are devised to preserve dentin matrix proteins.^[92]^ Although there exist techniques for complete removal of the organic matrix, their osteogenic potential is compromised due to the possible depletion of these valuable proteins.^[93]^ The primary objective is to maintain the biological potency of the graft while ensuring its safety and ease of use. The chairside preparation of ADM involves a meticulous process that carefully balances the dual goals of graft efficacy and safety. The resulting product is a versatile graft material that harnesses the inherent properties of dentin to facilitate bone regeneration.^[91]^

### 4.4. Demineralization reagents

Demineralization of dentin plays a pivotal role in its preparation as a graft material for diverse clinical applications. Varied demineralization reagents exert distinct effects on the organic and inorganic matrices of dentin.^[94,95]^

#### 4.4.1. Chelating agents

- Ethylenediaminetetraacetic acid (EDTA): A widely employed chelating agent in dentistry, particularly in endodontics for the purpose of removing smear layer. When utilized as a demineralizing agent, it selectively binds to calcium ions, resulting in the elimination of hydroxyapatite, the primary mineral constituent of dentin. The notable advantage associated with EDTA lies in its specificity; it predominantly targets the inorganic component while largely preserving the organic matrix. Consequently, this yields a collagen-rich scaffold that fosters favorable cellular adhesion and proliferation.^[96]^

#### 4.4.2. Strong acids

- 0.6N-HCL (hydrochloric acid): a potent acid capable of effectively demineralizing dentin. However, the utilization of strong acids necessitates precise regulation of exposure duration and concentration to prevent excessive harm to the organic matrix.^[97]^
- 2% HNO_3_ (nitric acid): Similar to HCL, nitric acid exhibits strong demineralization properties. However, careful control is necessary to prevent potential denaturation of the collagen component while effectively removing mineral content.^[72]^

#### 4.4.3. Weak acids

- Acetic acid: being a weak acid, exhibits a milder demineralization effect compared to strong acids. It enables the creation of a graded demineralization effect, facilitating controlled removal of the mineral phase while minimizing impact on the organic matrix.^[98]^
- Anthranilic acid: the demineralizing effects and impacts on the organic matrix of this weak acid are comparatively less aggressive when compared to strong acids.^[99]^

The extent of demineralization and potential alteration of the organic matrix is influenced by the concentration, duration of exposure, and specific properties of the chosen reagent.^[34,100]^ Given the absence of a universally accepted protocol for autogenous dentin demineralization, both clinicians and researchers often rely on empirical evidence and tailor their choice of demineralizing solution to suit the specific requirements of their procedures.^[100]^ The primary objective of the demineralization process should be to generate a scaffold that maintains its structural integrity while promoting cellular infiltration, adhesion, and subsequent osteogenesis.^[89]^

In conclusion, the meticulous selection of a demineralization agent, considering its potency and duration of application, plays a pivotal role in determining the quality and effectiveness of demineralized dentin for grafting purposes. When appropriately processed, demineralized dentin can exhibit exceptional osteoconductive properties along with potential osteoinductive characteristics, establishing it as an indispensable tool in bone regenerative treatments.^[101]^

### 4.5. Demineralization time

An extended demineralization process can result in the degradation of crucial proteins and growth factors associated with osteogenesis. Moreover, it may compromise the structural integrity of the dentin collagen fiber network, leading to increased graft resorption after implantation and impeding successful bone formation.^[63]^ Koga T’s investigation on dentin from a healthy population provided valuable insights into the extent of demineralization.^[102]^ At postoperative weeks 4 and 8, both groups exhibited some new bone formation based on microscopic CT and histological observations; however, the partially demineralized group demonstrated a greater extent of new bone formation. This finding suggests that complete demineralization may cause significant damage to essential components of the organic matrix required for new bone creation. In contrast, partial demineralization effectively eliminated most inorganic and immunogenic components while retaining crucial growth factors and bone-forming proteins.^[18]^ As a result, the bone-forming efficacy of demineralized teeth significantly improved. Undemineralized dentin exhibited higher resorption post-implantation compared to its partially demineralized counterpart. Although both types could induce some degree of new bone development, electron microscopic observations revealed that osteoblasts were unable to attach to undemineralized dentin due to insufficient exposure of the collagen fiber network, which serves as an osteoconductive scaffold for osteoblast attachment. The unexposed collagen fiber network and smaller diameter of dentin tubules limited the release of growth factors associated with osteogenesis.^[81]^ The balance between bone resorption and osteogenesis rates was found to be superior in partially demineralized dentin, particularly when its particle size was 1000 μm.^[102]^

In conclusion, demineralization plays a pivotal role in the release of biologically active components. The extent and duration of demineralization are crucial factors in dentin preparation for grafting. Achieving partial demineralization, which strikes a balance between preserving essential growth factors and proteins while enhancing the osteoconductive properties of the graft material, appears to be the most effective approach for bone regeneration.

## 5. Clinical application

### 5.1. Alveolar ridge preservation

Following tooth extraction, the absence of periodontal ligaments commonly leads to resorption of the alveolar bone and thinning of the labial bone plate. The inevitable occurrence of alveolar ridge contraction post-extraction has been extensively documented in literature, highlighting its clinical significance.^[103]^ In a recent randomized controlled clinical trial, Elfana et al compared both radiographic and histologic outcomes of autogenous whole-tooth grafts with autogenous demineralized dentin grafts for alveolar ridge preservation following tooth extraction in human subjects. The study confirmed the effectiveness of autogenous demineralized dentin grafts in maintaining volume, demonstrating positive results in histologic/histomorphometric analyses and reporting a low incidence of complications.^[34,104]^ Based on other reports, ADM also demonstrates comparable primary stability to those utilizing xenograft granules.^[105]^ ADM grafts present a potential alternative for vertical alveolar ridge augmentation.^[106–109]^

### 5.2. Maxillary sinus floor augmentation

In 2003, Murata^[108]^ reported the first clinical case of using an ADM graft in a sinus procedure. Subsequent follow-up studies confirmed exceptional bone formation. Ge’s research employed autogenous dental bone powder to address distal mesial bone defects in the posterior maxillary teeth.^[74]^ The findings underscored the treatment’s safety and efficacy in rectifying periodontal anomalies. When utilizing the same powder for maxillary sinus floor augmentation, a significant fusion of the boundary between the base of the maxillary sinus and alveolar bone was observed. The seamless integration of autogenous dental bone powder particles with newly formed bone within the sinus occurred. Bone growth initiated from the alveolar region, gradually ascending towards the sinus. This highlights the unique capability of the powder to rejuvenate low-crystalline inorganic materials and type I collagen, showcasing its stability and prowess in bone generation.^[35,110–113]^

### 5.3. Implantation

The autogenous tooth bone graft, obtained chairside from extracted teeth, has been successfully utilized for immediate implant placement in fresh sockets with labial bone defects.^[36,114]^ The gaps between the implant and the labial bone wall, as well as any defects in the labial bone, were filled with acellular dermal matrix (ADM) powder to ensure sufficient support of buccal bone. Wang et al^[33]^ conducted a comparative study utilizing autogenous dentin powder and Bio-Oss bone powder. The objective of the study was to address the buccal side gap of the implant and subsequently evaluate changes in bone volume at 6-month intervals. The findings revealed no significant differences in marginal bone loss between the 2 groups at both time points examined. However, patient feedback highlighted a notable advantage of using autogenous dentin powder, as it resulted in reduced postoperative pain and swelling compared to the allograft bone group. Importantly, patients expressed equal satisfaction with the outcomes regardless of the graft material used. These results suggested that while both autogenous dentin powder and Bio-Oss bone powder exhibit similar osteogenic potential in immediate implant scenarios, the former may offer superior postoperative patient comfort.

### 5.4. Guided bone regeneration (GBR)

Extracted teeth affected by severe periodontitis, yet retaining a relatively intact dental hard tissue structure, present an opportunity for grafting. In a clinical trial conducted by Li from 2015 to 2017 involving 40 patients, the effectiveness of autogenous demineralized dentin graft (DDM) was compared to Bio-Oss for immediate implantation combined with GBR.^[37]^ Remarkably, even in sites with post-extraction periodontitis, DDM demonstrated comparable clinical and radiographic outcomes to traditional osseous powder when utilized for immediate implant placement.^[115]^ Besides, ADM grafts, initially introduced for GBR, demonstrated successful maintenance of the formed corticocancellous bone with a dental implant over an average 5-year follow-up.^[116]^

### 5.5. Combination with other materials

The utilization of a combination of bone tissue regeneration materials often leads to superior outcomes in bone augmentation compared to the use of a single material alone. Initially, autogenous dentin was combined with calcium sulfate plaster.^[117]^ Subsequently, it was further integrated with additional materials such as calcium phosphate ceramics, hydroxyapatite/β –tricalcium phosphate (HA/β-TCP), platelet-rich fibrin (PRF),^[118]^ Bio-Oss,^[119]^ and autogenous bone. ADM combined with recombinant human bone morphogenetic protein-2 has been shown to promote effective bone regeneration without resulting in complications in human subjects.^[120]^ When autogenous dentin bone powder was combined with PRF in tooth extraction sockets to facilitate the osseous regeneration process, a gradual resorption of the dentin particles accompanied by concurrent new bone formation within the augmented area was observed. Postoperatively, patients reported minimal discomfort, and consistent stability of subsequent implant placements was noted.^[38]^ In addition to its autonomous potential, the efficacy of autogenous non-demineralized dentin in alveolar bone grafting can be enhanced through co-administration with MSCs.^[121]^

Autogenous tooth bone graft remains a versatile and efficacious grafting material in various clinical scenarios, owing to its capacity for synergistic integration with other materials, thereby offering patients enhanced post-operative comfort and consistent outcomes.^[122]^ Autogenous tooth bone graft prepared chairside is as effective as other bone grafting materials.^[6]^ Further research endeavors are expected to augment our comprehension of optimal application protocols.

## 6. Conclusion and perspectives

ADM has many advantages:

- Ease of acquisition: dental procedures often produce dental waste which can be repurposed.
- Cost-effectiveness: dentin offers a cost-effective alternative to many other grafting materials.
- Minimally invasive: harvesting dentin is often less invasive than obtaining autologous bone.
- Biosafety: due to its acellular nature, there is a reduced risk of disease transmission and antigenic reactions.
- Osteoinductive potential: partially demineralized dentin showcases promising osteoinductive and osteoconductive abilities.

However, there is no universally applicable approach to its preparation. Moreover, ADM may not always be the optimal choice, particularly in cases of extensive bone loss or absence of available teeth for extraction. The selection of an ideal bone graft material should be customized based on the unique characteristics of each specific bone defect to maximize repair outcomes. It is crucial to conduct comprehensive long-term studies in order to substantiate the osteogenic efficacy of ADM.

## Author contributions

**Conceptualization:** Wen Luo.

**Data curation:** Huifang Kuang.

**Formal analysis:** Huifang Kuang.

**Writing – original draft:** Honglan Sun, Xiaoyunqing Yin, Chao Yang.

**Writing – review & editing:** Chao Yang.

## References

[1] De Bruyn H Raes S Matthys C Cosyn J. The current use of patient-centered/reported outcomes in implant dentistry: a systematic review. Clin Oral Implants Res. 2015;26:45–56. 26385620 10.1111/clr.12634

[2] Gupta A Felton DA Jemt T Koka S. Rehabilitation of edentulism and mortality: a systematic review. J Prosthodont. 2019;28:526–35. 29573048 10.1111/jopr.12792

[3] Myeroff C Archdeacon M. Autogenous bone graft: donor sites and techniques. J Bone Joint Surg Am. 2011;93:2227–36. 22159859 10.2106/JBJS.J.01513

[4] García-Gareta E Coathup MJ Blunn GW. Osteoinduction of bone grafting materials for bone repair and regeneration. Bone. 2015;81:112–21. 26163110 10.1016/j.bone.2015.07.007

[5] Traini T Piattelli A Caputi S. Regeneration of human bone using different bone substitute biomaterials. Clin Implant Dent Relat Res. 2015;17:150–62. 23682753 10.1111/cid.12089

[6] Mahardawi B Rochanavibhata S Jiaranuchart S Arunjaroensuk S Mattheos N Pimkhaokham A. Autogenous tooth bone graft material prepared chairside and its clinical applications: a systematic review. Int J Oral Maxillofac Surg. 2023;52:132–41. 35618639 10.1016/j.ijom.2022.04.018

[7] Dimitriou R Jones E McGonagle D Giannoudis PV. Bone regeneration: current concepts and future directions. BMC Med. 2011;9:66. 21627784 10.1186/1741-7015-9-66 PMC3123714

[8] Nkenke E Weisbach V Winckler E. Morbidity of harvesting of bone grafts from the iliac crest for preprosthetic augmentation procedures: a prospective study. Int J Oral Maxillofac Surg. 2004;33:157–63. 15050072 10.1054/ijom.2003.0465

[9] Greenwald AS Boden SD Goldberg VM Khan Y Laurencin CT Rosier RN; American Academy of Orthopaedic Surgeons. The Committee on Biological Implants. Bone-graft substitutes: facts, fictions, and applications. J Bone Joint Surg Am. 2001;83:98–103. 11712842 10.2106/00004623-200100022-00007

[10] Campana V Milano G Pagano E. Bone substitutes in orthopaedic surgery: from basic science to clinical practice. J Mater Sci Mater Med. 2014;25:2445–61. 24865980 10.1007/s10856-014-5240-2 PMC4169585

[11] Finkemeier CG. Bone-grafting and bone-graft substitutes. J Bone Joint Surg Am. 2002;84:454–64. 11886919 10.2106/00004623-200203000-00020

[12] Oryan A Alidadi S Moshiri A Maffulli N. Bone regenerative medicine: classic options, novel strategies, and future directions. J Orthop Surg Res. 2014;9:18. 24628910 10.1186/1749-799X-9-18 PMC3995444

[13] Orsini G Ricci J Scarano A. Histologic and ultrastructural analysis of regenerated bone in maxillary sinus augmentation using a porcine bone-derived biomaterial. J Periodontol. 2000;71:1411–22. 10.1902/jop.2006.060181 17209782

[14] Dorozhkin SV. Calcium orthophosphate-based biocomposites and hybrid biomaterials. J Mater Sci. 2009;44:2343–87.

[15] Triplett RG Nevins M Marx RE. Pivotal, randomized, parallel evaluation of recombinant human bone morphogenetic protein-2/absorbable collagen sponge and autogenous bone graft for maxillary sinus floor augmentation. J Oral Maxillofac Surg. 2009;67:1947–60. 19686934 10.1016/j.joms.2009.04.085

[16] Giannoudis PV Dinopoulos H Tsiridis E. Bone substitutes: an update. Injury. 2005;36:S20–7. 16188545 10.1016/j.injury.2005.07.029

[17] Zhao R Yang R Cooper PR Khurshid Z Shavandi A Ratnayake J. Bone grafts and substitutes in dentistry: a review of current trends and developments. Molecules. 2021;26:3007. 34070157 10.3390/molecules26103007 PMC8158510

[18] Kim YK Kim SG Byeon JH. Development of a novel bone grafting material using autogenous teeth. Oral Surg Oral Med Oral Pathol Oral Radiol Endod. 2010;109:496–503. 20060336 10.1016/j.tripleo.2009.10.017

[19] Bhattacharjya C Gadicherla S Kamath AT. Tooth-derived bone graft material. World J Dent. 2016;7:32–5.

[20] Um IW Kim YK Mitsugi M. Autogenous demineralized dentin matrix from extracted tooth for the augmentation of alveolar bone defect: a prospective randomized clinical trial in comparison with anorganic bovine bone. Clin Oral Implants Res. 2017;28:809–15. 27279547 10.1111/clr.12885

[21] Agarwal R García AJ. Biomaterial strategies for engineering implants for enhanced osseointegration and bone repair. Adv Drug Deliv Rev. 2015;94:53–62. 25861724 10.1016/j.addr.2015.03.013 PMC4598264

[22] Valtanen RS Yang YP Gurtner GC Maloney WJ Lowenberg DW. Synthetic and bone tissue engineering graft substitutes: what is the future? Injury. 2021;52:S72–7. 32732118 10.1016/j.injury.2020.07.040

[23] Venkataiah VS Yahata Y Kitagawa A. Clinical applications of cell-scaffold constructs for bone regeneration therapy. Cells. 2021;10:2687. 34685667 10.3390/cells10102687 PMC8534498

[24] Wang T Guo Y. The host response to autogenous, allogeneic, and xenogeneic treated dentin matrix/demineralized dentin matrix oriented tissue regeneration. Tissue Eng Part B Rev. 2023;30:74–81. 37440326 10.1089/ten.TEB.2023.0065

[25] Fernández RF Bucchi C Navarro P Beltrán V Borie E. Bone grafts utilized in dentistry: an analysis of patients’ preferences. BMC Med Ethics. 2015;16:71. 26486125 10.1186/s12910-015-0044-6 PMC4618514

[26] Kim Y Nowzari H Rich SK. Risk of prion disease transmission through bovine-derived bone substitutes: a systematic review. Clin Implant Dent Relat Res. 2013;15:645–53. 22171533 10.1111/j.1708-8208.2011.00407.x

[27] Nasr S Slot DE Bahaa S Dörfer CE Fawzy El-Sayed KM. Dental implants combined with sinus augmentation: what is the merit of bone grafting? A systematic review. J Craniomaxillofac Surg. 2016;44:1607–17. 27622972 10.1016/j.jcms.2016.06.022

[28] Schwartz Z Mellonig JT Carnes DL. Ability of commercial demineralized freeze-dried bone allograft to induce new bone formation. J Periodontol. 1996;67:918–26. 8884650 10.1902/jop.1996.67.9.918

[29] Cervera-Maillo JM Morales-Schwarz D Morales-Melendez H Mahesh L Calvo-Guirado JL. Autologous tooth dentin graft: a retrospective study in humans. Medicina (Kaunas). 2021;58:56. 35056364 10.3390/medicina58010056 PMC8778028

[30] Özkahraman N Balcioğlu NB Soluk Tekkesin M Altundağ Y Yalçin S. Evaluation of the efficacy of mineralized dentin graft in the treatment of intraosseous defects: an experimental in vivo study. Medicina (Kaunas). 2022;58:103. 35056411 10.3390/medicina58010103 PMC8777758

[31] Kim ES Lee IK Kang JY Lee E-Y. Various autogenous fresh demineralized tooth forms for alveolar socket preservation in anterior tooth extraction sites: a series of 4 cases. Maxillofac Plast Reconstr Surg. 2015;37:27. 26366388 10.1186/s40902-015-0026-0 PMC4559089

[32] Mahardawi B Kyaw TT Mattheos N Pimkhaokham A. The clinical efficacy of autogenous dentin blocks prepared chairside for alveolar ridge augmentation: a systematic review and meta-analysis. Clin Oral Implants Res. 2023;34:1025–37. 37461220 10.1111/clr.14131

[33] Wang W Jiang Y Wang D Mei D Xu H Zhao B. Clinical efficacy of autogenous dentin grafts with guided bone regeneration for horizontal ridge augmentation: a prospective observational study. Int J Oral Maxillofac Surg. 2022;51:837–43. 34924269 10.1016/j.ijom.2021.06.012

[34] Elfana A El-Kholy S Saleh HA Fawzy El-Sayed K. Alveolar ridge preservation using autogenous whole-tooth versus demineralized dentin grafts: a randomized controlled clinical trial. Clin Oral Implants Res. 2021;32:539–48. 33565656 10.1111/clr.13722

[35] Jun SH Ahn JS Lee JI Ahn K-J Yun P-Y Kim Y-K. A prospective study on the effectiveness of newly developed autogenous tooth bone graft material for sinus bone graft procedure. J Adv Prosthodont. 2014;6:528–38. 25551014 10.4047/jap.2014.6.6.528 PMC4279053

[36] Issa DR Nassar M Elamrousy W. Immediately placed implants using simvastatin and autogenous tooth graft combination in periodontally compromised sites: a randomized controlled clinical trial. Clin Oral Investig. 2024;28:210. 10.1007/s00784-024-05596-4 38467945

[37] Li P Zhu H Huang D. Autogenous DDM versus Bio-Oss granules in GBR for immediate implantation in periodontal postextraction sites: a prospective clinical study. Clin Implant Dent Relat Res. 2018;20:923–8. 30230681 10.1111/cid.12667

[38] Yüceer-Çetiner E Özkan N Önger ME. Effect of autogenous dentin graft on new bone formation. J Craniofac Surg. 2021;32:1354–60. 33405453 10.1097/SCS.0000000000007403

[39] Kim YK Kim SG Oh JS. Analysis of the inorganic component of autogenous tooth bone graft material. J Nanosci Nanotechnol. 2011;11:7442–5. 22103215 10.1166/jnn.2011.4857

[40] Zhang S Li X Qi Y. Comparison of autogenous tooth materials and other bone grafts. Tissue Eng Regen Med. 2021;18:327–41. 33929713 10.1007/s13770-021-00333-4 PMC8169722

[41] Becker J Schuppan D Benzian H. Immunohistochemical distribution of collagens types IV, V, and VI and of pro-collagens types I and III in human alveolar bone and dentine. J Histochem Cytochem. 1986;34:1417–29. 3772076 10.1177/34.11.3772076

[42] Butler WT Mikulski A Urist MR Bridges G Uyeno S. Noncollagenous proteins of a rat dentin matrix possessing bone morphogenetic activity. J Dent Res. 1977;56:228–32. 265954 10.1177/00220345770560030601

[43] Moradian-Oldak J George A. Biomineralization of enamel and dentin mediated by matrix proteins. J Dent Res. 2021;100:1020–9. 34151644 10.1177/00220345211018405 PMC8381691

[44] Butler WT Brunn JC Qin C. Dentin extracellular matrix (ECM) proteins: comparison to bone ECM and contribution to dynamics of dentinogenesis. Connect Tissue Res. 2003;44:171–8. 12952193

[45] Ritchie HH Ritchie DG Wang LH. Six decades of dentinogenesis research. historical and prospective views on phosphophoryn and dentin sialoprotein. Eur J Oral Sci. 1998;106:211–20. 9541228 10.1111/j.1600-0722.1998.tb02178.x

[46] Jágr M Eckhardt A Pataridis S Mikšík I. Comprehensive proteomic analysis of human dentine. Eur J Oral Sci. 2012;120:259–68. 22813215 10.1111/j.1600-0722.2012.00977.x

[47] Chun SY Lee HJ Choi YA. Analysis of the soluble human tooth proteome and its ability to induce dentin/tooth regeneration. Tissue Eng Part A. 2011;17:181–91. 20695775 10.1089/ten.TEA.2010.0121

[48] Park ES Cho HS Kwon TG. Proteomics analysis of human dentin reveals distinct protein expression profiles. J Proteome Res. 2009;8:1338–46. 19193101 10.1021/pr801065s

[49] Lee MH Kim YJ Kim HJ. BMP-2-induced Runx2 expression is mediated by Dlx5, and TGF-beta 1 opposes the BMP-2-induced osteoblast differentiation by suppression of Dlx5 expression. J Biol Chem. 2003;278:34387–94. 12815054 10.1074/jbc.M211386200

[50] Lee KS Kim HJ Li QL. Runx2 is a common target of transforming growth factor beta1 and bone morphogenetic protein 2, and cooperation between Runx2 and Smad5 induces osteoblast-specific gene expression in the pluripotent mesenchymal precursor cell line C2C12. Mol Cell Biol. 2000;20:8783–92. 11073979 10.1128/mcb.20.23.8783-8792.2000 PMC86511

[51] Mansukhani A Bellosta P Sahni M Basilico C. Signaling by fibroblast growth factors (FGF) and fibroblast growth factor receptor 2 (FGFR2)-activating mutations blocks mineralization and induces apoptosis in osteoblasts. J Cell Biol. 2000;149:1297–308. 10851026 10.1083/jcb.149.6.1297 PMC2175120

[52] Hughes FJ Turner W Belibasakis G Martuscelli G. Effects of growth factors and cytokines on osteoblast differentiation. Periodontol 2000. 2006;41:48–72. 16686926 10.1111/j.1600-0757.2006.00161.x

[53] Dimitriou R Tsiridis E Giannoudis PV. Current concepts of molecular aspects of bone healing. Injury. 2005;36:1392–404. 16102764 10.1016/j.injury.2005.07.019

[54] Avery SJ Sadaghiani L Sloan AJ Waddington RJ. Analysing the bioactive makeup of demineralised dentine matrix on bone marrow mesenchymal stem cells for enhanced bone repair. Eur Cell Mater. 2017;34:1–14. 28692113 10.22203/eCM.v034a01

[55] Carvalho VA Tosello Dde O Salgado MA. Histomorphometric analysis of homogenous demineralized dentin matrix as osteopromotive material in rabbit mandibles. Int J Oral Maxillofac Implants. 2004;19:679–86. 15508983

[56] Nastase MV Young MF Schaefer L. Biglycan: a multivalent proteoglycan providing structure and signals. J Histochem Cytochem. 2012;60:963–75. 22821552 10.1369/0022155412456380 PMC3527886

[57] Fulmer MT Ison IC Hankermayer CR Constantz BR Ross J. Measurements of the solubilities and dissolution rates of several hydroxyapatites. Biomaterials. 2002;23:751–5. 11771695 10.1016/s0142-9612(01)00180-6

[58] Lu J Descamps M Dejou J. The biodegradation mechanism of calcium phosphate biomaterials in bone. J Biomed Mater Res. 2002;63:408–12. 12115748 10.1002/jbm.10259

[59] Kim YK Kim SG Yun PY. Autogenous teeth used for bone grafting: a comparison with traditional grafting materials. Oral Surg Oral Med Oral Pathol Oral Radiol. 2014;117:e39–45. 22939321 10.1016/j.oooo.2012.04.018

[60] Priya A Nath S Biswas K Basu B. In vitro dissolution of calcium phosphate-mullite composite in simulated body fluid. J Mater Sci Mater Med. 2010;21:1817–28. 20411309 10.1007/s10856-010-4053-1

[61] Kim SJ Kim MR Oh JS Han I Shin S-W. Effects of polycaprolactone-tricalcium phosphate, recombinant human bone morphogenetic protein-2 and dog mesenchymal stem cells on bone formation: pilot study in dogs. Yonsei Med J. 2009;50:825–31. 20046425 10.3349/ymj.2009.50.6.825 PMC2796411

[62] Chae HS Choi H Park I Moon YS Sohn DS. Comparative histomorphometric analysis of bone regeneration according to bone graft type. Int J Oral Maxillofac Implants. 2023;38:1191–9. 38085751 10.11607/jomi.10312

[63] Li J Yang J Zhong X He F Wu X Shen G. Demineralized dentin matrix composite collagen material for bone tissue regeneration. J Biomater Sci Polym Ed. 2013;24:1519–28. 23848446 10.1080/09205063.2013.777227

[64] Duraine G Hu J Athanasoiu K. Bioengineering in the oral cavity: insights from articular cartilage tissue engineering. Int J Oral Maxillofac Implants. 2011;26:11–9; discussion 20. 21464997 PMC3164493

[65] Lu HH Subramony SD Boushell MK Zhang X. Tissue engineering strategies for the regeneration of orthopedic interfaces. Ann Biomed Eng. 2010;38:2142–54. 20422291 10.1007/s10439-010-0046-y PMC3665605

[66] Mafi P Hindocha S Mafi R Khan WS. Evaluation of biological protein-based collagen scaffolds in cartilage and musculoskeletal tissue engineering—a systematic review of the literature. Curr Stem Cell Res Ther. 2012;7:302–9. 22563667 10.2174/157488812800793045

[67] Retzepi M Donos N. Guided bone regeneration: biological principle and therapeutic applications. Clin Oral Implants Res. 2010;21:567–76. 20666785 10.1111/j.1600-0501.2010.01922.x

[68] Kolbeck S Bail H Weiler A Windhagen H Haas N Raschke M. Digital radiography. A predictor of regenerate bone stiffness in distraction osteogenesis. Clin Orthop Relat Res. 1999;366:221–8. 10627739

[69] Aghaloo TL Moy PK. Which hard tissue augmentation techniques are the most successful in furnishing bony support for implant placement? Int J Oral Maxillofac Implants. 2007;22:49–70. 18437791

[70] Murata M Sato D Hino J. Acid-insoluble human dentin as carrier material for recombinant human BMP-2. J Biomed Mater Res A. 2012;100:571–7. 22213638 10.1002/jbm.a.33236

[71] Murata M Kabir MA Hirose Y. Histological evidences of autograft of dentin/cementum granules into unhealed socket at 5 months after tooth extraction for implant placement. J Funct Biomater. 2022;13:66. 35735921 10.3390/jfb13020066 PMC9224646

[72] Grawish ME Grawish LM Grawish HM. Demineralized dentin matrix for dental and alveolar bone tissues regeneration: an innovative scope review. Tissue Eng Regen Med. 2022;19:687–701. 35429315 10.1007/s13770-022-00438-4 PMC9294090

[73] Um IW. Demineralized dentin matrix (DDM) as a carrier for recombinant human bone morphogenetic proteins (rhBMP-2). Adv Exp Med Biol. 2018;1077:487–99. 30357705 10.1007/978-981-13-0947-2_26

[74] Catanzaro-Guimarães SA Catanzaro-Guimarães B Garcia RB. Osteogenic potential of autogenic demineralized dentin implanted in bony defects in dog. Int J Oral Maxillofac Surg. 1986;15:160–9. 3083019 10.1016/s0300-9785(86)80136-3

[75] Lee HJ Hong JS Kim YK Um I-W Lee J-I. Osteogenic potential of demineralized dentin matrix as bone graft material. J Hard Tissue Biol. 2017;26:223–30.

[76] MaTanoue R Ohta K Miyazono Y. Three-dimensional ultrastructural analysis of the interface between an implanted demineralised dentin matrix and the surrounding newly formed bone. Sci Rep. 2018;8:858. 29434259 10.1038/s41598-018-21291-3 PMC5809602

[77] Gomes MF dos Anjo MJ Nogueira TO. Histologic evaluation of the osteoinductive property of autogenous demineralized dentin matrix on surgical bone defects in rabbit skulls using human amniotic membrane for guided bone regeneration. Int J Oral Maxillofac Implants. 2001;16:563–71. 11516004

[78] Saito T Toyooka H Ito S Crenshaw MA. In vitro study of remineralization of dentin: effects of ions on mineral induction by decalcified dentin matrix. Caries Res. 2003;37:445–9. 14571124 10.1159/000073398

[79] Bessho K Tanaka N Matsumoto J Tagawa T Murata M. Human dentin-matrix-derived bone morphogenetic protein. J Dent Res. 1991;70:171–5. 1999554 10.1177/00220345910700030301

[80] Wang W Li X Mei D Zhao B. Autogenous solid dentin for horizontal ridge augmentation with simultaneous implantation in a severe bone defect: a 3.5-year follow-up clinical report. J Prosthet Dent. 2023:S0022-3913(23)00412-2. 10.1016/j.prosdent.2023.05.036 37442750

[81] Ike K Urist MR. Recycled dentin root matrix for a carrier of recombinant human bone morphogenetic protein. J Oral Implantol. 1998;24:124–32. 9893518 10.1563/1548-1336(1998)024<0124:RDRMFA>2.3.CO;2

[82] Kim KW. Bone Induction by demineralized dentin matrix in nude mouse muscles. Maxillofac Plast Reconstr Surg. 2014;36:50–6. 27489810 10.14402/jkamprs.2014.36.2.50 PMC4281903

[83] Kim YK Lee JH Kim KW Um I-W Murata M Ito K. Analysis of organic components and osteoinductivity in autogenous tooth bone graft material. J Korean Assoc Maxillofac Plast Reconstr Surg. 2013;35:353–9.

[84] Kim YK Lee JK Kim KW. Chapter 16: Healing mechanism and clinical application of autogenous tooth bone graft material. In: Pignatello R, ed., Advances in biomaterials science and biomedical applications. Rijeka: Intech. 2013:405.

[85] Murata M Akazawa T Hino J. Biochemical and histo-morphometrical analyses of bone and cartilage induced by human decalcified dentin matrix and BMP-2. Oral Biol Res. 2011;35:9–14.

[86] Rijal G Shin HI. Human tooth-derived biomaterial as a graft substitute for hard tissue regeneration. Regen Med. 2017;12:263–73. 28350271 10.2217/rme-2016-0147

[87] Um IW Lee JK Kim JY. Allogeneic dentin graft: a review on its osteoinductivity and antigenicity. Materials (Basel). 2021;14:1713. 33807291 10.3390/ma14071713 PMC8036611

[88] Lei G Wang Y Yu Y. Dentin-derived inorganic minerals promote the osteogenesis of bone marrow-derived mesenchymal stem cells: potential applications for bone regeneration. Stem Cells Int. 2020;2020:8889731. 33293964 10.1155/2020/8889731 PMC7691015

[89] Kim ES. Autogenous fresh demineralized tooth graft prepared at chairside for dental implant. Maxillofac Plast Reconstr Surg. 2015;37:8. 25705613 10.1186/s40902-015-0009-1 PMC4331600

[90] Kim YK Lee J Um IW. Tooth-derived bone graft material. J Korean Assoc Oral Maxillofac Surg. 2013;39:103–11. 24471027 10.5125/jkaoms.2013.39.3.103 PMC3858164

[91] Kim YK Kim SG Bae JH Um I-W Oh J-S Jeong K-I. Guided bone regeneration using autogenous tooth bone graft in implant therapy: case series. Implant Dent. 2014;23:138–43. 24637527 10.1097/ID.0000000000000046

[92] Barón M Morales V Fuentes MV Linares M Escribano N Ceballos L. The influence of irrigation solutions in the inorganic and organic radicular dentine composition. Aust Endod J. 2020;46:217–25. 31984636 10.1111/aej.12395

[93] Besinis A van Noort R Martin N. Remineralization potential of fully demineralized dentin infiltrated with silica and hydroxyapatite nanoparticles. Dent Mater. 2014;30:249–62. 24444789 10.1016/j.dental.2013.11.014

[94] Lippert F Churchley D Lynch RJ. Effect of lesion baseline severity and mineral distribution on remineralization and progression of human and bovine dentin caries lesions. Caries Res. 2015;49:467–76. 26228732 10.1159/000431039

[95] Miller CA Ashworth E Deery C El Sharkasi L Moorehead RD Martin N. Effect of demineralising agents on organic and inorganic components of dentine. Caries Res. 2021;55:521–33. 34348278 10.1159/000518463

[96] Gandolfi MG Taddei P Pondrelli A Zamparini F Prati C Spagnuolo G. Demineralization, collagen modification and remineralization degree of human dentin after EDTA and citric acid treatments. Materials (Basel). 2018;12:25. 30577625 10.3390/ma12010025 PMC6337713

[97] Isik AG Tarim B Hafez AA Yalçin FS Onan U Cox CF. A Comparative scanning electron microscopic study on the characteristics of demineralized dentin root surface using different tetracycline HCl concentrations and application times. J Periodontol. 2000;71:219–25. 10711612 10.1902/jop.2000.71.2.219

[98] Sousa SM Silva TL. Demineralization effect of EDTA, EGTA, CDTA and citric acid on root dentin: a comparative study. Braz Oral Res. 2005;19:188–92. 16308606 10.1590/s1806-83242005000300006

[99] Ikemura K Arai K Hashimoto H Kawakami T. Effects of aminobenzoic acid derivatives with 4-AET/HEMA in self-etching primer on bonding to ground dentin. Dent Mater J. 1996;15:144–53. 9550012 10.4012/dmj.15.144

[100] Koga T Minamizato T Kawai Y. Bone regeneration using dentin matrix depends on the degree of demineralization and particle size. PLoS One. 2016;11:e0147235. 26795024 10.1371/journal.pone.0147235 PMC4721666

[101] Cammack GV Nevins M Clem DS Hatch JP Mellonig JT. Histologic evaluation of mineralized and demineralized freeze-dried bone allograft for ridge and sinus augmentations. Int J Periodontics Restorative Dent. 2005;25:231–7. 16001735

[102] Gomes MF Abreu PP Morosolli ARC Araújo MM Goulart MGV. Densitometric analysis of the autogenous demineralized dentin matrix on the dental socket wound healing process in humans. Braz Oral Res. 2006;20:324–30. 17242793 10.1590/s1806-83242006000400008

[103] Araújo MG Lindhe J. Dimensional ridge alterations following tooth extraction. An experimental study in the dog. J Clin Periodontol. 2005;32:212–8. 15691354 10.1111/j.1600-051X.2005.00642.x

[104] Parvini P Schliephake C Al-Maawi S. Histomorphometrical assessment of vertical alveolar ridge augmentation using extracted tooth roots in the canine. Clin Oral Investig. 2020;24:317–23. 10.1007/s00784-019-02960-7 31102042

[105] Lee JY Kim YK Yi YJ Choi J-H. Clinical evaluation of ridge augmentation using autogenous tooth bone graft material: case series study. J Korean Assoc Oral Maxillofac Surg. 2013;39:156–60. 24471036 10.5125/jkaoms.2013.39.4.156 PMC3858125

[106] Kim YK Yun PY Um IW. Alveolar ridge preservation of an extraction socket using autogenous tooth bone graft material for implant site development: prospective case series. J Adv Prosthodont. 2014;6:521–7. 25551013 10.4047/jap.2014.6.6.521 PMC4279052

[107] de Oliveira GS Miziara MN Silva ER Ferreira EL Biulchi APF Alves JB. Enhanced bone formation during healing process of tooth sockets filled with demineralized human dentine matrix. Aust Dent J. 2013;58:326–32. 23981214 10.1111/adj.12088

[108] Murata M. Autogenous demineralized dentin matrix for maxillary sinus augmentation in humans. In the first clinical report; proceedings of the 81st international association for dental research, Gothenburg, Sweden. 2003; IADR/PER General Session.

[109] Ge J Yang C Zheng J Hu Y. Autogenous bone grafting for treatment of osseous defect after impacted mandibular third molar extraction: a randomized controlled trial. Clin Implant Dent Relat Res. 2017;19:572–80. 27933720 10.1111/cid.12466

[110] Kim YK Jun SH Um IW Kim S. Evaluation of the healing process of autogenous tooth bone graft material nine months after sinus bone graft: micromorphometric and histological evaluation. Maxillofac Plast Reconstr Surg. 2013;35:310–5.

[111] Oliveira ER Nie L Podstawczyk D. Advances in growth factor delivery for bone tissue engineering. Int J Mol Sci. 2021;22:903. 33477502 10.3390/ijms22020903 PMC7831065

[112] Umebayashi M Ohba S Kurogi T Noda S Asahina I. Full regeneration of maxillary alveolar bone using autogenous partially demineralized dentin matrix and particulate cancellous bone and marrow for implant-supported full arch rehabilitation. J Oral Implantol. 2020;46:122–7. 31910061 10.1563/aaid-joi-D-19-00315

[113] Wu D Zhou L Lin J Chen J Huang W Chen Y. Immediate implant placement in anterior teeth with grafting material of autogenous tooth bone vs xenogenic bone. BMC Oral Health. 2019;19:266. 31791302 10.1186/s12903-019-0970-7 PMC6889614

[114] Taschieri S Morandi B Alberti A. Immediate implant positioning using tooth-derived bone substitute material for alveolar ridge preservation: preliminary results at 6 months. Clin Exp Dent Res. 2023;9:17–24. 36366869 10.1002/cre2.685 PMC9932247

[115] Kim SG Kim HK Lim SC. Combined implantation of particulate dentine, plaster of paris, and a bone xenograft (Bio-Oss) for bone regeneration in rats. J Craniomaxillofac Surg. 2001;29:282–8. 11673923 10.1054/jcms.2001.0236

[116] Minamizato T Koga T Takashi I. Clinical application of autogenous partially demineralized dentin matrix prepared immediately after extraction for alveolar bone regeneration in implant dentistry: a pilot study. Int J Oral Maxillofac Surg. 2018;47:125–32. 28802762 10.1016/j.ijom.2017.02.1279

[117] Flanagan D. Autogenous dentin with calcium sulfate as graft material: a case series. J Oral Implantol. 2022;48:285–94. 34170327 10.1563/aaid-joi-D-20-00309

[118] Alrmali A Saleh MHA Mazzocco J Zimmer JM Testori T Wang H-L. Auto-dentin platelet-rich fibrin matrix is an alternative biomaterial for different augmentation procedures: a retrospective case series report. Clin Exp Dent Res. 2023;9:993–1004. 37933487 10.1002/cre2.808 PMC10728516

[119] Kim YK Lee JH Um IW Cho W-J. Guided bone regeneration using demineralized dentin matrix: long-term follow-up. J Oral Maxillofac Surg. 2016;74:515.e1–9. 10.1016/j.joms.2015.10.030 26679551

[120] Kim SY Kim YK Park YH. Evaluation of the healing potential of demineralized dentin matrix fixed with recombinant human bone morphogenetic protein-2 in bone grafts. Materials (Basel). 2017;10:1049. 28880245 10.3390/ma10091049 PMC5615704

[121] Barreiro BOB Koth VS Sesterheim P. Autogenous dentin combined with mesenchymal stromal cells as an alternative alveolar bone graft: an in vivo study. Clin Oral Investig. 2023;27:1907–22. 10.1007/s00784-022-04840-z 36574044

[122] Gomes MF dos Anjos MJ Nogueira Tde O. Autogenous demineralized dentin matrix for tissue engineering applications: radiographic and histomorphometric studies. Int J Oral Maxilofac Implants. 2002;17:488–97. 12182291

